# Comparative gene expression analysis in closely related dermatophytes reveals secondary metabolism as a candidate driver of virulence

**DOI:** 10.1128/spectrum.01383-25

**Published:** 2025-10-09

**Authors:** Lenka Machová, Martin Kostovčík, Karel Švec, Vít Hubka, Miroslav Kolařík, Adéla Wennrich

**Affiliations:** 1 Laboratory of Fungal Genetics and Metabolism, Institute of Microbiology, Czech Academy of Sciences 86863, Prague, Czech Republic; 2 Department of Genetics and Microbiology, Faculty of Science, Charles University, Prague, Czech Republic; Universidade de Brasilia, Brasilia, Federal District, Brazil

**Keywords:** *ex vivo* skin model, RT-qPCR, virulence factors, gene expression, secondary metabolites, *Trichophyton benhamiae* complex, dermatophytes

## Abstract

Dermatophytes are important fungal skin pathogens affecting humans and animals worldwide. Although several virulence factors have been identified using genomic, proteomic, and transcriptomic approaches, their roles remain incompletely understood. In this study, we applied a comparative approach using four closely related taxa within the *Trichophyton benhamiae* complex, which differ in infectivity despite sharing common hosts. We focused on the emerging zoonotic pathogen *T.benhamiae* var. *luteum*, currently responsible for epidemic outbreaks in Europe, and compared it to its less infective relatives. A set of 16 candidate genes, informed by preliminary transcriptomic screening, was assessed via RT-qPCR across 12 strains grown *in vitro* (Sabouraud dextrose broth) and *ex vivo* (murine skin explants). Genes associated with secondary metabolism were consistently upregulated under *ex vivo* conditions, particularly in *T.benhamiae* var. *luteum*. While two of the biosynthetic gene clusters examined are linked to known metabolites, others remain uncharacterized. These findings reveal key gene expression differences that may explain the enhanced infectivity of emerging strains and underscore the potential role of secondary metabolites in dermatophyte virulence. They also highlight the need for improved genome annotation in *T.benhamiae* to better understand the molecular basis of pathogenesis.

**IMPORTANCE:**

*Trichophyton benhamiae* var. *luteum* is an emerging fungal pathogen responsible for a rising number of skin infections transmitted from guinea pigs to humans, especially in Europe. We investigated why this pathogen spreads more effectively than its close relatives, which infect the same hosts but are less epidemic. Using a laboratory model that mimics skin infection, we found that genes involved in producing fungal compounds—called secondary metabolites, some of which act as toxins—are more active in this pathogen. These compounds may help the fungus suppress the host immune response and establish infection. Our findings shed light on how fungal pathogens adapt to their hosts and highlight gene pathways that could be targeted in future diagnostics or treatments. Understanding these mechanisms is key to managing emerging fungal threats in both animals and humans.

## INTRODUCTION

Dermatophytes are widespread fungal pathogens with a prevalence of 20%–25% in the world’s human population (1). In recent decades, awareness of these fungi has grown due to the rise in antifungal-resistant strains and the discovery of new species (2–4). One major public health issue is the rapid spread of *Trichophyton benhamiae* var. *luteum*, commonly referred to as “yellow-phenotype strains” of *T.benhamiae* in Europe. This emerging zoonotic pathogen has spread extensively in European guinea pig farms and pet shops, and among their owners and their children, especially young individuals (4–6). Interestingly, closely related species, *T.japonicum* and *T.europaeum* (commonly referred to as “white-phenotype strains”), have been present on the same hosts in Europe for decades without triggering similar outbreaks and epidemics (4). Coinfection of guinea pigs with both *T.benhamiae* var. *luteum* and “white-phenotype strains” (i.e., *T.europaeum* and *T.japonicum*) has been documented; however, the epidemic strains of *T.benhamiae* var. *luteum* cause human infections up to 30 times more frequently and up to four times more frequently in guinea pigs than its closely related species (4, 6, 7). One explanation for the lower incidence of *T.japonicum* and *T.europaeum* compared with *T. benhamiae* var. *luteum* in guinea pig and human populations may be their lower infectivity. In addition, a population of a different variety (*T.benhamiae* var. *benhamiae*) belonging to the same species as the epidemic strains (*T.benhamiae* var. *luteum*) occurs in North America, yet no reports of epidemics caused by these strains have been documented in that area (4). It remains unclear why the epidemic strain spreads so effectively among European guinea pigs and children compared with its close relatives or even other populations of the same species.

Dermatophytes have developed various mechanisms for spreading within hosts and invading their tissues. A key feature is their sophisticated machinery for degrading keratin and other skin components, primarily through the secretion of proteases. Notable examples include subtilisin-like proteases, dipeptidyl peptidases, leucine aminopeptidases, and metallopeptidases (8, 9). Hosts counteract dermatophyte infections using several defense mechanisms, such as high body temperature, and the mildly acidic pH of the skin, as well as innate immunity like keratinocytes, macrophages, neutrophils, and adaptive immunity, especially the Th1 and Th17 cell-mediated response (10, 11). Dermatophytes can evade some host barriers and produce a wide range of proteases that vary according to the environmental pH (12–15). As a result of keratin degradation by dermatophytes, the pH of the surrounding area increases, which impairs the host’s natural immunity (14, 16). Compounds produced by dermatophytes, such as penicillin G, melanin, xanthomegnin, and vioxanthin are known or suspected toxins and/or immunomodulators (17–20). Nevertheless, the role of secondary metabolites in dermatophyte pathogenesis remains underexplored.

Despite the high genetic similarity among dermatophytes (4, 21), different species exhibit distinct host preferences and varying levels of virulence across host species. These differences likely arise from variations in gene expression and enzyme activity regulation (22). Several approaches can be used to identify potential virulence factors. In dermatophytes, comparative studies have primarily focused on gene expression during *in vitro* saprobic and *in vivo* parasitic growth phases (23, 24). Various alternatives to the *in vivo* approach have been introduced as useful, more ethical, cost-effective, and reproducible options (25). Several *ex vivo* skin models have been developed in addition to the traditional *in vitro* models to better replicate real conditions of infection in dermatophytes (26–31). These include reconstructed human epidermis (30) and organoid-based models (31), which offer human tissue relevance but may lack full skin architecture, immune elements, or mechanical barrier integrity. In contrast, animal-derived explants (26–29) preserve native skin structure and allow short-term fungal colonization under near-physiological conditions. Another option is the comparison of related species differing in infectivity or virulence. However, this requires the use of very closely related populations or species to ensure that the observed results are not simply attributed to differences between species during their speciation. This comparative approach has not been used for dermatophytes.

In this study, we applied a comparative gene expression approach to identify candidate virulence-associated genes in dermatophytes. We compared more infective taxa with less infective, yet closely related ones to identify differences in gene expression potentially linked to virulence. This approach allows us to distinguish general virulence factors shared across taxa from those potentially associated with enhanced infectivity. Candidate genes were selected based on preliminary transcriptomic data and literature evidence, and their expression was then validated across a broader strain data set using RT-qPCR to strengthen the robustness of observed patterns.

## MATERIALS AND METHODS

### Strains and cultivation conditions

A total of 12 strains—three from each of the four closely related taxa, *T.benhamiae* var. *benhamiae*, *T.benhamiae* var. *luteum*, *T.japonicum*, and *T.europaeum—*were used (Table 1). All strains are publicly available from the Culture Collection of Fungi, Charles University (CCF). Species identification was confirmed using ITS rDNA sequencing (4). Cultures were maintained on malt extract agar (MEA; HiMedia) at 6 °C in the dark. Prior to the experiment, all strains were subcultured to ensure purity. To minimize the risk of contamination, a single-colony isolation step was performed by spreading diluted inoculum on MEA plates and selecting a single morphologically typical colony for further use. Strains were also briefly cultured in liquid medium supplemented with chloramphenicol, and bacterial contamination was ruled out by microscopic inspection.

**TABLE 1:** Strains used in the gene expression analysis

Taxon	Referred to as	Strain ID^*a*^	CCF identifier	Year of isolation	Source
*T.benhamiae* var. *benhamiae*	a	IHEM 4710 ^T^	CCF6484	1956	Human skin
	b	USA 3356	CCF6486	2010	Dog
	c	IHEM 3287	CCF6483	1970	Monoascosporic isolate no. 3 from crossing of strains IHEM 24908× IHEM 4710
*T.benhamiae* var. *luteum*	a	IHEM 25742	CCF6474	2012	Human skin
	b	SK 1248/12	CCF4852	2012	Skin near mouth of a 6-year-old girl
	c	IHEM 25077	CCF6475	2011	Human scalp and neck skin
*T.europaeum*	a	IHEM 20161 = CBS 112371	CCF6479	2002	Face of human (after contact with guinea pigs)
	b	IHEM 25062	CCF6477	2011	Face of human
	c	ME 192/12	CCF6380	2012	Thigh skin of a 16-year-old girl
*T.japonicum*	a	IHEM 17701^T^	CCF6481	1963	Human skin
	b	VUT 97010	CCF6489	1997	Rabbit
	c	NUBS 13002	CCF6488	Unknown	Human skin

^*a*^ Superscript “T” denotes ex-type strains. Abbreviation of the culture collections: IHEM-BCCM/IHEM - Biomedical Fungi and Yeast Collection, Belgium; USA-University of Illinois at Urbana-Champaign, USA; SK-Clinic of Dermatovenerology, General University Hospital in Prague, Czech Republic; ME-Hospital in Pardubice, Czech Republic; VUT-Nihon University School of Veterinary Medicine, Japan; NUBS-Nihon University School of Veterinary Medicine, Japan.

To prepare the inoculum, strains were grown in Sabouraud dextrose broth (SDB; HiMedia) at 30 °C for 8 days, shaking at 200  rpm (Digital Orbital Shaker, Heathrow Scientific). The resulting biomass was homogenized by vortexing and diluted to obtain approximately equal cell densities. This method was used instead of standard spore suspensions because some strains—particularly *T.benhamiae* var. *luteum*—were sterile.

Three to five biological replicates were prepared for each strain and for both cultivation conditions: liquid culture and *ex vivo* skin model. Liquid cultures were initiated by inoculating 30 mL of SDB with 4 µL of homogenate and incubated under the same conditions described above.

### Murine skin models

The *ex vivo* murine skin explant (MSE) model protocol was developed and optimized based on published methodologies (31–33). Healthy three-month-old male BALB/c mice were obtained from the breeding facility of the Institute of Microbiology, CAS. All procedures involving animals were conducted in full compliance with Czech legislation (Act No. 246/1992 Coll., on the protection of animals against cruelty) and the Guidelines for the Care and Use of Laboratory Animals.

The dorsal skin of live mice was shaved using an electric clipper (Aesculap Exacta GT 416; B. Braun, Germany), and the animals were subsequently euthanized. The shaved area was swabbed with 70% ethanol. Skin sections (2 × 2  cm) were excised under sterile conditions, cleaned of adipose tissue, and placed into 0.1% benzalkonium bromide solution for disinfection.

Within 30 minutes, samples were washed with ice-cold phosphate-buffered saline (PBS; Sigma-Aldrich), immersed in 1% Penicillin-Streptomycin (Pen-Strep; Sigma-Aldrich) for 10 minutes, then washed twice again with PBS and Pen-Strep. Finally, skin samples were placed on sterile gauze in 6-well plates.

Each sample received 1  mL of murine skin fluid (MSF), composed of 10% (v/v) heat-inactivated fetal bovine serum (Gibco), 8% (v/v) MSF buffer, and 1% (v/v) Pen-Strep. MSF buffer (adjusted to pH 6.4) contained: 8.0  g/l NaCl, 0.4  g/l KCl, 0.0875  g/l Na₂HPO₄, 0.0625  g/l KH₂PO₄, 0.2  g/l MgSO₄, and 6.25  g/l D-glucose.

Four microliters of SDB culture supernatant, microscopically confirmed to be free of bacterial contamination, was added to each skin sample. The samples were incubated at 30 °C and 5% CO₂ for 8 days without shaking (MCO-170AICUV-PE incubator, Panasonic). MSF was refreshed daily. The 8-day cultivation period was chosen based on a pilot RNAseq analysis.

### RNA extraction and processing

Mycelia from liquid cultures were harvested by centrifugation at 1000  rpm for 10 minutes. Mycelium or colonized skin was ground in liquid nitrogen, resuspended in 1  mL of TRIzol reagent (Thermo Fisher Scientific), and stored at –35 °C until RNA isolation. RNA was extracted following the manufacturer’s instructions (34). Equal amounts of total RNA were used for downstream cDNA synthesis, but fungal biomass was not quantified separately from host tissue in *ex vivo* samples.

RNA concentration and purity were assessed using a NanoDrop 1000 spectrophotometer (Thermo Fisher Scientific). Genomic DNA was removed with the TURBO DNA-free Kit and protected using RNaseOUT Recombinant Ribonuclease Inhibitor (both Thermo Fisher Scientific). RNA integrity was confirmed by 1% agarose gel electrophoresis stained with ethidium bromide (HiMedia). Concentration was measured using Qubit RNA HS and BR Assay Kits on a Qubit 2.0 Fluorometer (Thermo Fisher Scientific).

### Candidate gene identification by RNA sequencing

To identify genes potentially involved in virulence and host interaction, RNA-seq was performed on two representative strains—*T*.*benhamiae* var. *luteum* (IHEM 25742) and *T.japonicum* (NUBS 13002)— under both *in vitro* and *ex vivo* conditions.

RNA quality was further verified using the Agilent 2100 Bioanalyzer and the RNA 6000 Nano Kit (Agilent Technologies). Due to moderate RNA degradation (RIN <8), library preparation was adjusted accordingly.

Ribosomal RNA was depleted using the NEBNext Poly(A) mRNA Magnetic Isolation Module (New England Biolabs), and libraries were prepared with the KAPA RNA HyperPrep Kit (Roche). Twenty nanograms of RNA was used for input; fragmentation was performed at 85 °C for 2 minutes, followed by 14 amplification cycles. Indexed adapters (KAPA Dual-Indexed Adapter Kit, Roche) were used, and sequencing was performed on the Illumina NextSeq 500 platform.1

Reads were aligned using STAR v.2.7.1 (35) and mapped with Bowtie2 (36) to a genome index based on *T.europaeum* strain CBS 112371 (RefSeq: GCF_000151125.1). Differential expression analysis was conducted in R v.3.6.0 using the DESeq2 package (37), with standard error and log₂ fold change computed. The Wald test and Benjamini-Hochberg correction were applied. All replicates from both conditions were used. Genes with adjusted *P*-values < 0.05 were considered significant and annotated using NCBI and UniProt databases.

### RT-qPCR analysis

For RT-qPCR, 1  µg of total RNA was reverse-transcribed using the LunaScript RT SuperMix Kit (New England Biolabs) according to the manufacturer’s protocol. Complementary DNA (cDNA) from three high-quality biological replicates was pooled in equal amounts prior to qPCR. This approach was chosen to minimize noise from individual-level variation and to ensure sufficient cDNA quantity across the large panel of target genes and conditions. We acknowledge that pooling limits the ability to assess within-strain variability.

Quantitative PCR was performed using the CFX96 Real-Time System (Bio-Rad Laboratories) and the Luna Universal qPCR Master Mix (New England Biolabs). The PCR reaction was done with 0.5 µL of 10 pM primers, and 0.5 µL of cDNA in the final volume of 20 µL. The reaction comprised an initial denaturation at 95 °C for 60 s, followed by 39 cycles of 95 °C for 15 s and 60 °C for 30 s, inactivation at 95 °C for 60 s, cooldown at 54 °C for 60 s, and final cooldown at 25 °C for 60 s. Melting curve analysis was conducted by increasing the temperature from 54 °C to 95 °C in 0.5 °C increments every 10 s. Each measurement was performed in duplicate, and if the standard deviation exceeded 2, the experiment was repeated.

### Primer design and efficiency testing

Primer pairs for ARB_00701 and TERG_04919 were adapted from previously published studies (23, 38). All other primers were designed using Primer-BLAST (NCBI) based on the genome sequence of *T.europaeum* CBS 112371 (RefSeq: GCF_000151125.1). Each primer pair was designed such that at least one primer spanned an exon–exon junction to avoid amplification of genomic DNA.

The OligoAnalyzer tool (IDT) was used to assess potential secondary structures, with accepted primers showing ΔG > –7 J for dimers and > –4 J for hairpins. All primer pairs were tested using pooled cDNA and serial dilutions (1:10; 1:50; 1:250; 1:1,250; 1:6,250). Primer efficiencies ranged from 85% to 100% (Supplemental material 2a).

### RT-qPCR data processing and analysis

The stability and reliability of four previously published reference genes were analyzed using RefFinder (39), which integrates the tools BestKeeper (40), NormFinder (41), and geNorm (42). Specifically, the analyzed genes were ARB_06116 (DNA-directed RNA polymerase II subunit RPB2*; rpb2*) (43), ARB_03667 (ADP-ribosylation factor; *adp-rf*) (44, 45), ARB_00512 (Mitotic cohesin complex subunit Psm1; *psm1*) (44, 45), and TERG_04919 (Chitin synthase; CHS) (46). Based on this analysis, *rpb2* and *adp-rf* were selected as the most stable reference genes (Supplemental material 2b).

For processing of the acquired RT-qPCR data, the software Bio-Rad CFX Manager v. 3.1 (Bio-Rad Laboratories) was used. Due to the large number of genes examined and the inability to measure the expression of all genes on a single plate for both conditions, the efficiency-weighted method (47) was chosen for data normalization. For the same reason, both reference genes were always run on each plate for all measured samples as an interplate calibrator. The 2^-ΔΔCt^ analysis was performed. As technical replicates were used and cDNA was pooled prior to qPCR, statistical comparisons reflect between-strain variation rather than intra-strain variability. The cycle threshold (Ct) range, response efficiency, coefficient of determination, relative gene expression (ΔCt) values for each strain under different culture conditions, and the effect size measure values (2^-ΔΔCt^) for individual genes are provided in Supplemental material 3. The normal distribution of data was tested with the use of the Shapiro-Wilk test. Based on the results, either ANOVA or Kruskal-Wallis tests were performed, followed by pairwise *t*-tests. Results are shown in Supplemental material 4.

For the statistical analysis, R v. 4.2.1 (48) was used with the package stats, reshape2 (49), FactoMineR (50), and factoextra (51). The visualization of data was performed with the use of packages from the Tidyverse (52), especially ggplot2 (53), and the packages viridis (54), gridExtra (55), and cowplot (56).

### Bioinformatic identification of Biosynthetic Gene Clusters

Biosynthetic gene clusters (BGCs) in the genome of *T.benhamiae* were identified using the fungal version of antiSMASH v.7.1.0 (57). To assess the level of similarity of genes or clusters of interest, the Ident and Sim tool (https://www.bioinformatics.org/sms2/ident_sim.htmL), BLAST v. 2.15.0 (58), and MAFFT (59) were used. The visualization of clusters was performed in R v. 4.2.1 (48) with the gggenes package (60).

#### Histological analysis

Prior to RNA extraction, 5 × 5 mm sections of selected skin samples were fixed, embedded, and stained. Fixation was performed in 4% paraformaldehyde and 10% formalin for 48 h (61). Samples were dehydrated using a Leica ASP200s and embedded in paraffin using a Leica EG110 system.

Sections (3 µm) were cut using a Leica RM2125 RTS rotary microtome and placed on precoated slides (Electron Microscopy Sciences). Staining was performed using the standard hematoxylin and eosin (H&E) protocol (62–64), with an additional benzene clearing step. Samples were mounted in Canadian balsam, covered with glass coverslips, and left to harden overnight. H&E-stained sections were used to qualitatively confirm fungal invasion and tissue colonization. No quantitative fungal burden measurement (e.g., by ITS copy number or CFU counts) was performed.

## RESULTS

### Selection of target genes

**Fig 1:**
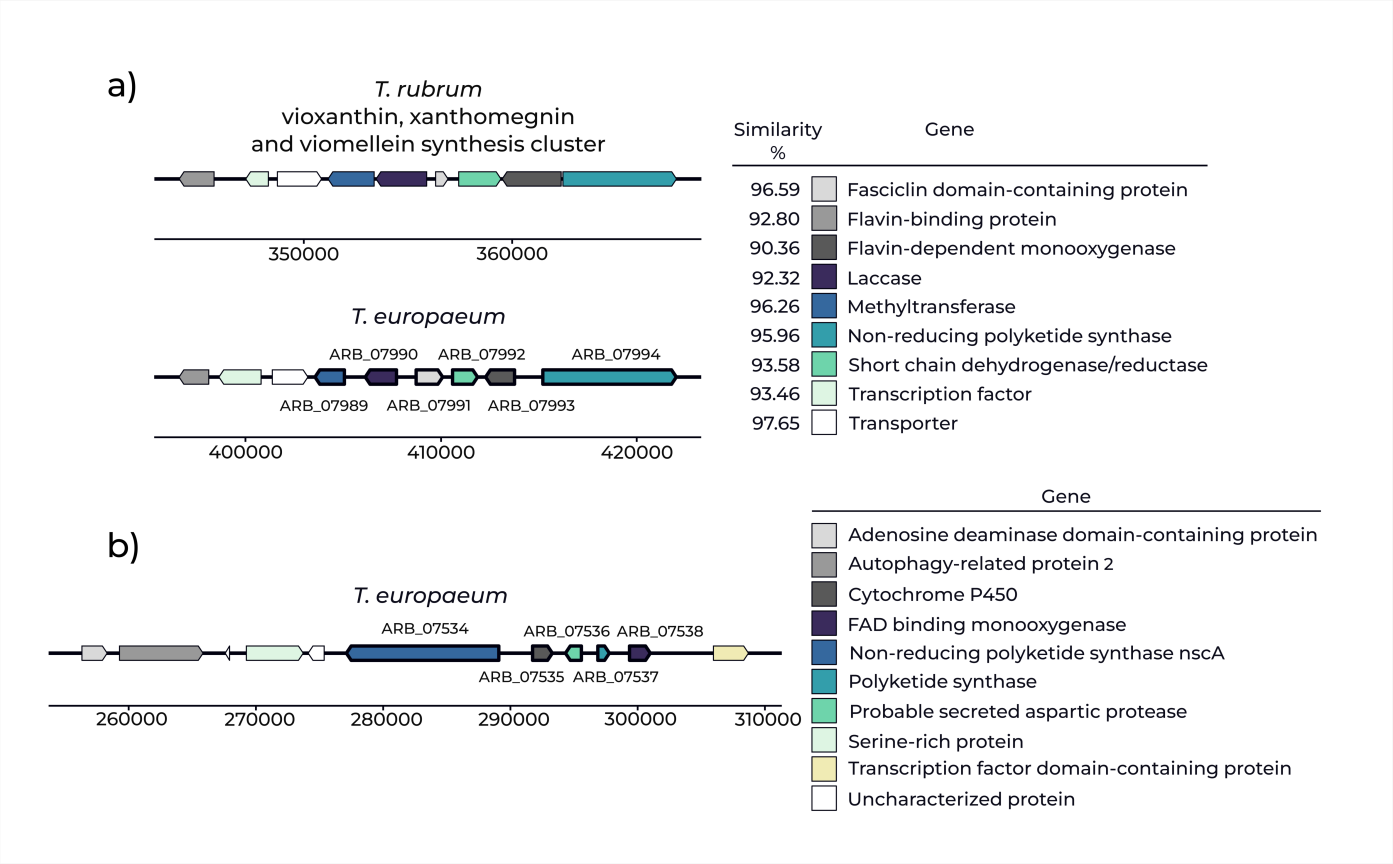
Biosynthetic gene clusters (BGCs) related to secondary metabolism as predicted by antiSMASH analysis. (**a**) Cluster A: biosynthetic gene cluster responsible for the synthesis of vioxanthin, xanthomegnin, and viomellein in *Trichophyton rubrum* (strain CBS 118892), compared with the genome of *T.europaeum* (strain CBS 112371); similarity values are based on BLAST analysis. (**b**) Cluster B: uncharacterized biosynthetic gene cluster in *T.europaeum* with an unknown product. Genes that showed significant differential expression in RNA-seq analysis are annotated and highlighted with bold outlines.

A total of 85 genes were significantly differentially expressed (adjusted *P* < 0.05) between *T.benhamiae* var. *luteum* IHEM 25742 and *T.japonicum* NUBS 13002. The putative functions of more than half were inferred from the predicted protein structure. According to antiSMASH analysis, 11 genes belonged to two biosynthetic gene clusters (BGCs). One cluster was identified as the vioxanthin, xanthomegnin, and viomellein BGC and is referred to here as cluster A; the second, of unknown function, is referred to as cluster B (Table 2; Fig. 1). Additionally, two other genes (ARB_04645, ARB_07966) were identified in the data set (Table 2). ARB_04645 is a component of a previously described ergot alkaloid synthesis pathway in Arthrodermataceae (65). The cluster associated with ARB_07966 could not be identified. Other differentially expressed genes were associated with transmembrane transport, basic cellular functions, cell wall remodeling, and sulfur metabolism (see Supplemental material 1 for the full list of differentially expressed genes).

**TABLE 2:** Results of RNA-seq analysis performed using the DESeq2 package, comparing *T.benhamiae* var. *luteum* and *T.japonicum^a^*

Locus	Log2 fold change	Adjusted *P*-value	Annotation NCBI	Annotation UniProt	Possible function
ARB_06975	−13.80	5.27 × 10^−12^	Hydrophobin, putative	Hydrophobin A	Surface hydrophobicity, adherence
ARB_05303	6.26	5.84 × 10^−08^	Uncharacterized protein	Sulfotransferase family protein	Sulfur-related processes
ARB_00847	−11.16	3.45 × 10^−07^	Uncharacterized protein	Uncharacterized protein	–^*b*^
ARB_07538	6.84	2.12 × 10^−06^	FAD binding monooxygenase, putative	FAD binding monooxygenase, putative	Secondary metabolism, cluster B
ARB_03989	−10.49	1.56 × 10^−05^	Uncharacterized protein	Uncharacterized protein	–
ARB_04141	−8.46	4.18 × 10^−05^	Uncharacterized protein	Uncharacterized protein	–
ARB_01369	−10.71	7.33 × 10^−05^	Uncharacterized protein	Uncharacterized protein	–
ARB_07952	−8.10	0.00011882	Uncharacterized protein	Uncharacterized protein	–
ARB_05947	6.72	0.00012002	Uncharacterized protein	FAD-binding PCMH-type domain-containing protein	Undetermined
**ARB_07994**	**6.76**	**0.00032219**	**Conidial pigment polyketide synthase** **PksP/Alb1**	**Non-reducing polyketide synthase (NscA)**	**Secondary metabolism, cluster A**
ARB_04859	5.05	0.00033383	Oxalate decarboxylase, putative	Probable oxalate decarboxylase	Primary metabolism
ARB_02085	−9.14	0.00042272	Uncharacterized protein	Methyltransferase domain-containing protein	Various biochemical reactions
ARB_07989	7.98	0.00054269	O-methyltransferase, putative	O-methyltransferase, putative	Secondary metabolism, cluster A
ARB_07993	7.53	0.00054269	Uncharacterized protein	Uncharacterized protein	Secondary metabolism, cluster A
**ARB_07534**	**6.86**	**0.00066905**	**LovB-like polyketide synthase, putative**	**Non-reducing polyketide synthase (NscA)**	**Secondary metabolism, cluster B**
ARB_07518	−9.63	0.00069182	Uncharacterized protein	Uncharacterized protein	–
ARB_04430	−8.78	0.00069182	Uncharacterized protein	Uncharacterized protein	–
ARB_07536	8.80	0.00087444	Uncharacterized protein	Probable secreted aspartic protease	Secondary metabolism, cluster B
ARB_04594	8.38	0.00087444	Uncharacterized protein	STAS domain-containing protein	Sulfur-related processes
**ARB_03861**	**5.11**	**0.00087739**	**Glutathione S-transferase Ure2-like, putative**	**Glutathione S-transferase Ure2-like, putative**	**Stress-related processes**
ARB_07951	−8.30	0.00087739	Uncharacterized protein	DUF3328 domain protein	Undetermined
ARB_06429	−9.81	0.00087739	O-methyltransferase	O-methyltransferase	Various biochemical reactions
ARB_05141	−8.65	0.00087739	Uncharacterized protein	Uncharacterized protein	–
ARB_05392	−7.73	0.00098404	Uncharacterized protein	Chitinase	Cell wall-related processes
ARB_02932	6.79	0.00133298	RTA1 domain protein, putative	RTA1 domain protein, putative	Undetermined
ARB_07276	4.72	0.00157637	Uncharacterized protein	phosphoadenosine phosphosulfate reductase domain-containing protein	Sulfur*-*related processes
ARB_05274	4.96	0.00195658	Conserved uncharacterized protein	Uncharacterized protein	–
ARB_04644	−5.81	0.00208368	Uncharacterized protein	Lumazine-binding protein	Undetermined
ARB_06173	4.66	0.00208841	Zinc-containing alcohol dehydrogenase, putative	Zinc-containing alcohol dehydrogenase, putative	Undetermined
ARB_01072	7.31	0.00210063	Carboxylesterase, putative	Carboxylic ester hydrolase	Various biochemical reactions
ARB_03721	5.78	0.00256559	Uncharacterized protein	AB hydrolase-1 domain-containing protein	Undetermined
ARB_07517	−7.66	0.00270752	Uncharacterized protein	Acyl-protein thioesterase 1	Sulfur-related processes
ARB_07246	−9.05	0.00270752	Uncharacterized protein	DUF1203 domain protein	Undetermined
ARB_01258	−3.87	0.00301641	Uncharacterized protein	Uncharacterized protein	–
ARB_01444	−7.01	0.00329636	Uncharacterized protein	Glucan endo-1,3-beta-D-glucosidase	Cell wall-related processes
ARB_07535	7.25	0.00386691	Uncharacterized protein	Cytochrome P450	Secondary metabolism, cluster B
ARB_05140	−7.40	0.00396502	Uncharacterized protein	Protein kinase domain-containing protein	Basic cellular processes
ARB_01724	−5.24	0.0041556	Hsp90 binding co-chaperone (Sba1), putative	Hsp90 binding co-chaperone (Sba1), putative	Basic cellular processes
**ARB_07991**	**7.48**	**0.0041556**	**Fasciclin domain family protein**	**Fasciclin domain family protein**	**Secondary metabolism, cluster A**
ARB_04955	−7.43	0.0041556	Uncharacterized protein	Uncharacterized protein	–
ARB_01579	−7.22	0.00518502	Uncharacterized protein	Uncharacterized protein	–
ARB_07992	6.66	0.00613564	Short chain dehydrogenase/reductase family protein	Short chain dehydrogenase/reductase family protein	Secondary metabolism, cluster A
**ARB_03514**	**−7.93**	**0.0069251**	**Class III chitinase ChiA2**	**Class III chitinase**	**Cell wall-related processes**
ARB_00424	3.33	0.0069251	Uncharacterized protein	Glutathione transferase	Sulfur-related processes
ARB_05391	3.76	0.0069251	PTR family peptide transporter, putative	PTR family peptide transporter, putative	Transmembrane transport
ARB_00651	−7.26	0.00707818	Uncharacterized protein	HNH nuclease domain-containing protein	Undetermined
ARB_06241	−6.27	0.00867606	Opsin, putative	Opsin, putative	Undetermined
ARB_05812	4.24	0.00962227	Uncharacterized protein	Fatty acid hydroxylase domain-containing protein	Primary metabolism
ARB_03863	−7.33	0.01095632	Uncharacterized protein	Uncharacterized protein	–
ARB_01940	4.02	0.01095632	Homogentisate 1,2-dioxygenase, putative	Homogentisate 1,2-dioxygenase	Various biochemical reactions
ARB_03395	−4.26	0.01250736	Solid-state culture expressed protein (Aos23), putative	Solid-state culture expressed protein (Aos23), putative	Undetermined
ARB_06838	−6.83	0.01278189	GPI-anchored endo-1,3(4)-beta-glucanase, putative	GPI-anchored endo-1,3(4)-beta-glucanase, putative	Cell wall-related processes
**ARB_04645**	**−6.42**	**0.01278189**	**Uncharacterized protein**	**Catalase easC**	**Synthesis of ergot alkaloids**
ARB_01913	−7.08	0.01288591	Uncharacterized protein	Uncharacterized protein	–
ARB_02208	3.52	0.0133612	Uncharacterized protein	Oxalate decarboxylase	Primary metabolism
**ARB_07818**	**−3.32**	**0.0133612**	**Cytochrome P450 monooxygenase, putative**	**Cytochrome P450 monooxygenase, putative**	**Various biochemical reactions**
ARB_06800	−6.79	0.01489926	Uncharacterized protein	Uncharacterized protein	–
ARB_07966	3.61	0.0151062	Uncharacterized protein	Non-reducing polyketide synthase (NscA)	Secondary metabolism
ARB_01650	−5.56	0.0158866	Uncharacterized protein	Uncharacterized protein	–
ARB_00746	5.89	0.0158866	Uncharacterized protein	Major facilitator superfamily (MFS) profile domain- containing protein	Transmembrane transport
ARB_04467	−3.58	0.01673777	Glucanase, putative	Probable glucan 1,3-beta-glucosidase	Cell wall-related processes
ARB_04431	−6.92	0.01749836	Uncharacterized protein	altered inheritance of mitochondria protein 9, mitochondrial	Undetermined
ARB_01248	4.09	0.02039071	Zinc-containing alcohol dehydrogenase, putative	Zinc-containing alcohol dehydrogenase, putative	Undetermined
ARB_00248	−6.96	0.02259337	NAD-dependent epimerase/dehydratase family protein	NAD-dependent epimerase/dehydratase family protein	Undetermined
ARB_01027	3.10	0.02563679	Uncharacterized protein	MFS-type efflux pump MFS1	Transmembrane transport
ARB_07899	−6.84	0.02563679	Folylpolyglutamate synthetase, putative	Folylpolyglutamate synthase	Basic cellular processes
ARB_06251	−7.70	0.02846453	Uncharacterized protein	Uncharacterized protein	–
ARB_04058	−3.95	0.02918407	DNA repair and transcription factor Ada, putative	DNA repair and transcription factor Ada, putative	Basic cellular processes
ARB_07483	−3.10	0.02966119	Heat shock protein Awh11, putative	Heat shock protein Awh11, putative	Basic cellular processes
ARB_03753	5.25	0.02971309	Uncharacterized protein	Major facilitator superfamily (MFS) profile domain- containing protein	Transmembrane transport
ARB_02282	−6.74	0.03048244	Uncharacterized protein	Uncharacterized protein	–
ARB_06102	−6.47	0.03048244	Uncharacterized protein	Aminoglycoside phosphotransferase domain- containing protein	Various biochemical reactions
ARB_04769	6.71	0.03048244	Uncharacterized protein	Probable neutral protease 2 homolog	Secreted protease
ARB_05177	−6.92	0.03148407	Uncharacterized protein	Upregulated in Daf-2 domain-containing protein	Undetermined
ARB_03519	5.33	0.03163056	Uncharacterized protein	RTA1 domain protein	Undetermined
ARB_07381	−5.84	0.03260191	Uncharacterized protein	Uncharacterized protein	–
ARB_03167	−6.36	0.03381736	Uncharacterized protein	Uncharacterized protein	–
ARB_07537	6.21	0.03381736	Polyketide synthase, putative	Polyketide synthase, putative	Secondary metabolism, cluster B
ARB_06539	−7.51	0.03381736	Uncharacterized protein	Uncharacterized protein	–
**ARB_07990**	**5.99**	**0.03754576**	**Conidial pigment biosynthesis oxidase Arb2**	**Conidial pigment biosynthesis oxidase Arb2**	**Secondary metabolism, cluster A**
ARB_06768	−3.47	0.03754576	Phosphate-repressible phosphate permease, putative	Phosphate transporter	Transmembrane transport
ARB_05610	−6.21	0.03754576	Uncharacterized protein	Uncharacterized protein	–
ARB_03617	−2.59	0.03890598	Uncharacterized protein	LEA domain protein	Undetermined
ARB_05078	−5.59	0.04451843	Uncharacterized protein	Alpha-carbonic anhydrase domain-containing protein	Undetermined

^*a*^ The table displays the top 85 statistically significant genes (adjusted *P* < 0.05). Genes belonging to biosynthetic gene clusters (BGCs) investigated further are labeled as Cluster A or Cluster B. Genes selected for RT-qPCR validation are shown in bold.

^*b*^ "–” indicates not assigned.

**Fig 2:**
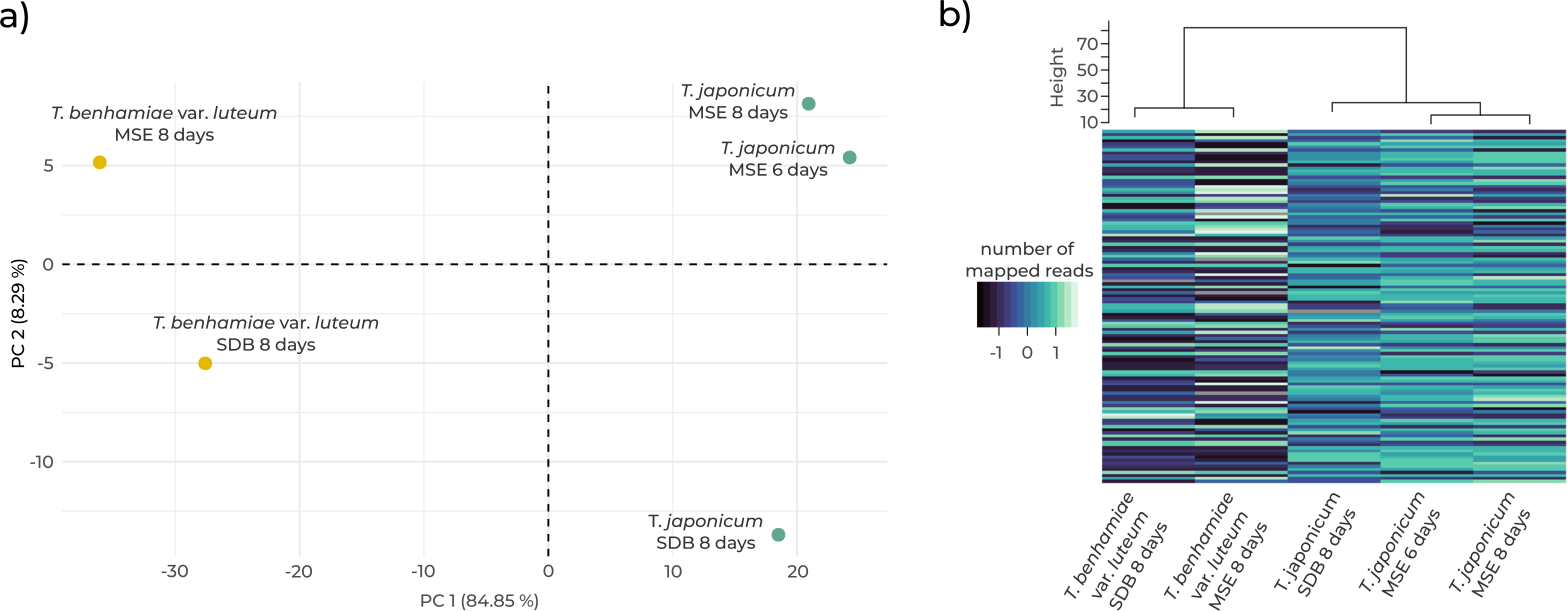
Transcriptomic analysis of epidemic (*T.benhamiae* var. *luteum*) and non-epidemic (*T.japonicum*) strains within the *T.benhamiae* complex. The figure shows the 85 most significantly differentially expressed genes identified from transcriptomic data. Strains were cultivated *in vitro* in Sabouraud dextrose broth (SDB) and *ex vivo* on murine skin explants (MSE). (**a**) Principal component analysis (PCA) of gene expression variation. (**b**) Ward’s hierarchical clustering based on expression similarity. Cultivation was terminated at 6 or 8 days post-inoculation.

Based on the expression profiles of the 85 significant genes, the samples were divided into two primary clusters that reflected strain identity (Fig. 2a). The first principal component (PC1) accounted for 85% of total variability, separating samples by species, while the second component (PC2) distinguished samples based on cultivation condition. A hierarchical clustering analysis also grouped the samples primarily by species (Fig. 2b). Samples of *T.japonicum* (NUBS 13002) collected at 6 and 8 days post-inoculation showed minimal variation in gene expression (Fig. 2).

From these data, 16 genes representing diverse cellular functions were selected for further investigation across a larger data set of 12 strains from the *T.benhamiae* complex using RT-qPCR on cDNA pooled from biological replicates (Table 3). Nine genes were selected from the transcriptomic data set—seven of which showed significant inter-strain differences, and five were associated with BGCs. Two additional genes (ARB_02741 and ARB_05770) were included due to their presumed functional relevance despite non-significant p-values. Another seven genes were selected based on a literature review as known dermatophyte virulence factors.

**TABLE 3:** List of genes analyzed by RT-qPCR, including predicted function or activity and the source literature upon which their selection was based^*a*^

Locus	Protein name	Function/activity	Source
ARB_00701	Subtilisin 3 (Sub3)	Keratinolytic protease (66), adherence (67)	(23, 24, 68)
ARB_06110	Dipeptidyl peptidase (DPP) IV	Keratinolytic protease (69)	(23, 24, 68, 69)
ARB_06651	Dipeptidyl peptidase (DPP) V	Keratinolytic protease (69)	(23, 24, 68–70)
ARB_00494	Leucin aminopeptidase 2 (Lap 2)	Keratinolytic protease (70)	(23, 24, 68, 69)
ARB_04170	Aspartic-type endopeptidase (OPSB)	Peptidase (71)	(24)
ARB_07638	Malate synthase	Glyoxylate cycle (72)	(23, 73)
ARB_07814	Isocitrate lyase	Glyoxylate cycle (72)	(23, 73)
ARB_03514	Class III chitinase ChiA2	Cell wall growth and remodeling (71)	RNAseq analysis
ARB_05770	1,3-Beta-glucanosyltransferase	Cell wall growth and remodeling (74)	RNAseq analysis
ARB_02741	GPI-anchored CFEM domain protein	Cell wall stability (71)	RNAseq analysis
ARB_07818	Cytochrome P450 monooxygenase	Metabolism (75)	RNAseq analysis
ARB_04645	Catalase easC	Ergot alkaloids synthesis (76)	RNAseq analysis
ARB_07994	Pigment polyketide synthase from cluster A	Vioxanthin, xanthomegnin, and viomellein synthesis (77, 78)	RNAseq analysis
ARB_07991	Fasciclin domain family protein (cluster A)	Vioxanthin, xanthomegnin, and viomellein synthesis (77, 78)	RNAseq analysis
ARB_07534	Polyketide synthase from cluster B	Secondary metabolism (57)	RNAseq analysis
ARB_03861	Glutathione S-transferase Ure2-like	Stress management (79)	RNAseq analysis

^*a*^ Protein annotations were obtained from the UniProt database.

### Functional annotation of selected genes

Bioinformatic analysis revealed that six genes from cluster A (ARB_07989–ARB_07994) share strong similarity with a laccase-containing vioxanthin, xanthomegnin, and viomellein BGC from *T. rubrum* (77, 78) (Fig. 1; Supplemental material 5). The five genes in cluster B (ARB_07534–ARB_07538) could not be linked to any known metabolite. The core gene (ARB_07534) was annotated as either a LovB-like polyketide synthase or a non-reducing polyketide synthase encoded by *nscA*. However, protein-level identity was only 21% with the lovastatin synthase LovB from *A. terreus* (ATEG_09961) (80) and 17.64% with the neosartoricin synthase NscA from *T. tonsurans* (TESG_06702) (81). Based on this low sequence similarity, it is unlikely that ARB_07534 performs the same biosynthetic function as either of these enzymes.

#### RT-qPCR analysis

### Gene expression under *in vitro* and *ex vivo* conditions

**Fig 3:**
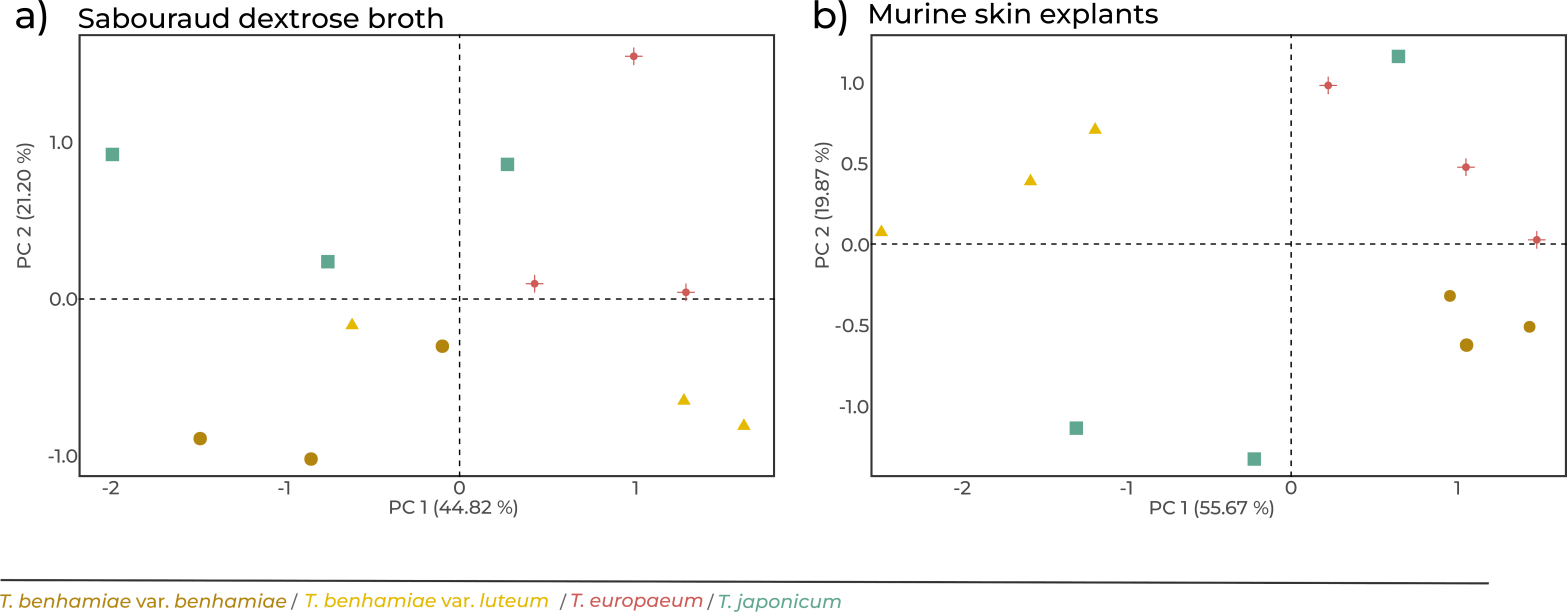
Principal component analysis (PCA) of *T.benhamiae* complex taxa based on ΔCt values from RT-qPCR data. Samples were obtained from *in vitro* SDB (**a**) and *ex vivo* MSE (**b**) cultivation.

In both cultivation systems, sample clustering reflected species-level differences, except in a few cases: *T.benhamiae* var. *benhamiae* vs. var. *luteum* under *in vitro* (SDB) conditions, and *T.japonicum* under *ex vivo* (MSE) conditions. In the *ex vivo* model, the species were more clearly separated, and even the two closely related *T.benhamiae* varieties were distinctly grouped (Fig. 3).

**Fig 4:**
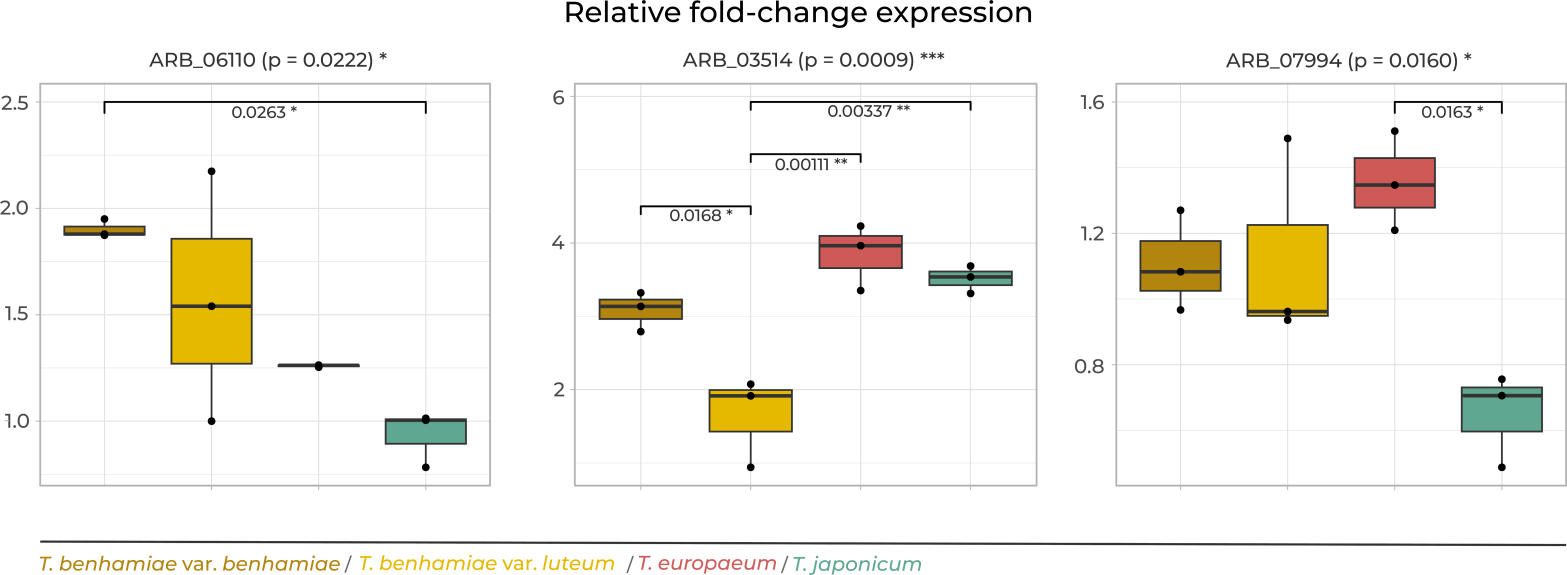
Relative gene expression fold changes (2^-ΔΔCt^) for genes with significant differential expression between *in vitro* (SDB) and *ex vivo* (MSE). Asterisks indicate statistical significance (*P < 0.05; **P < 0.01; ***P < 0.001).

Gene expression was generally higher under *ex vivo* conditions, but only three genes showed statistically significant differences (Supplemental material 3c and 4). The *DPPIV* protease gene (ARB_06110) showed significantly different expression between *T.benhamiae* var. *benhamiae* and *T.japonicum. T.europaeum* significantly overexpressed polyketide synthase ARB_07994 (cluster A) compared with *T.japonicum*. Additionally, expression of class III chitinase *chiA2* (ARB_03514) was significantly lower in *T.benhamiae* var. *luteum* than in the other taxa (Supplemental material 4; Fig. 4).

**Fig 5:**
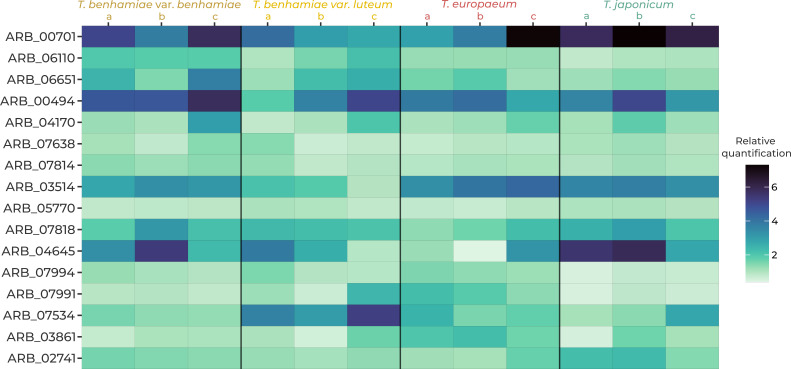
Heatmap of relative gene expression fold changes (2^-ΔΔCt^) for all measured genes under ex vivo (MSE) versus *in vitro* (SDB) conditions. Includes both statistically significant and non-significant genes.

Interestingly, several genes were consistently upregulated under *ex vivo* conditions across nearly all strains of the *T.benhamiae* complex. Notably, *SUB3* (ARB_00701) with up to sevenfold change, *LAP2* (ARB_00494) with up to fivefold change, Class III chitinase *chiA2* (ARB_03514) with up to fourfold change, cytochrome P450 monooxygenase (ARB_07818) with up to threefold change, and catalase *easC* (ARB_04645) with up to fivefold change (Fig. 5; Supplemental material 3c).

### Differential expression under *in vitro* conditions

**Fig 6:**
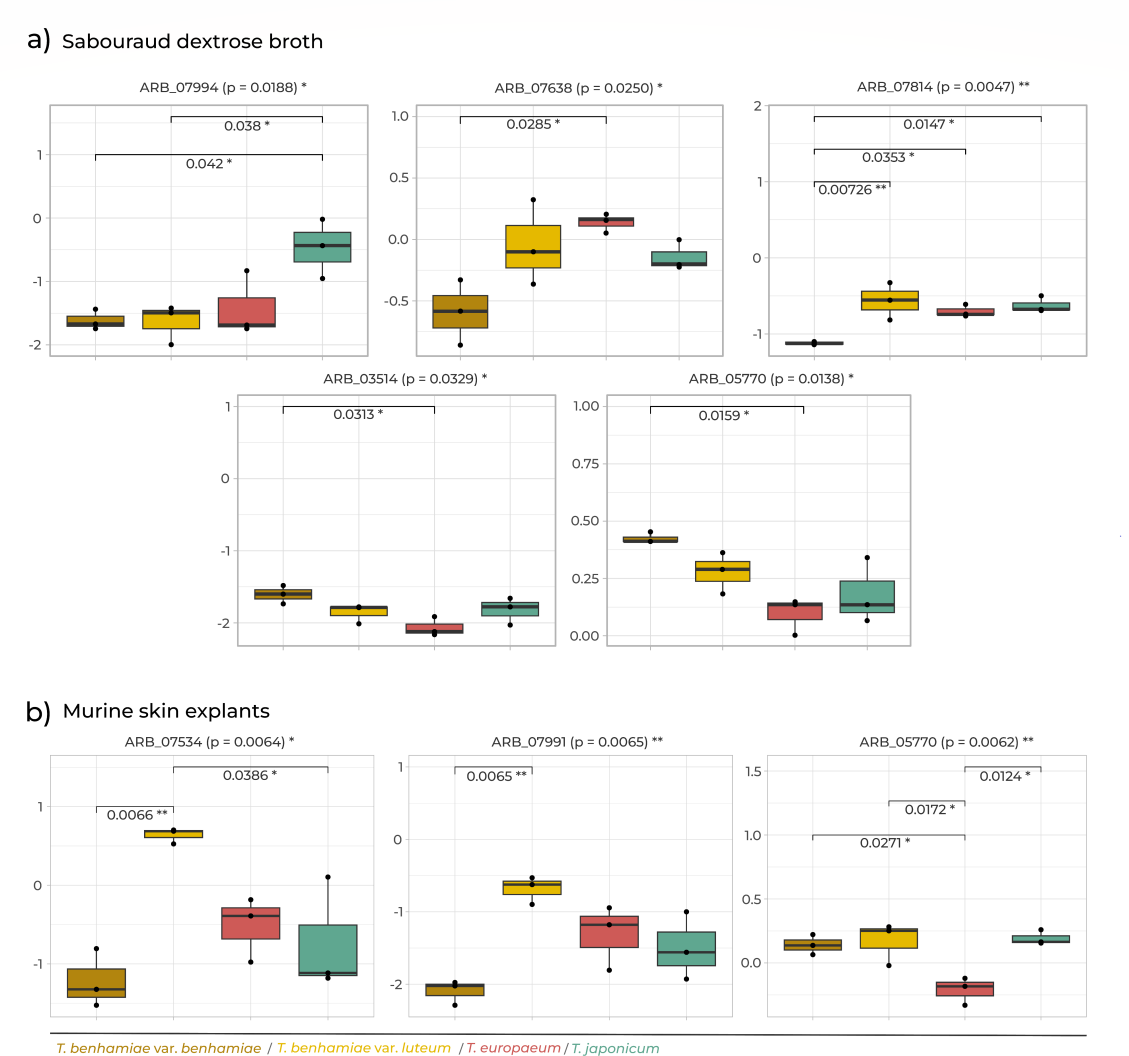
Expression of selected genes that differed significantly between taxa of the *T. benhamiae* complex when cultivated: (**a**) *in vitro* (SDB) and (**b**) *ex vivo* (MSE). Asterisks indicate statistical significance (*P < 0.05; **P < 0.01; ***P < 0.001).

Under *in vitro* conditions (SDB), moderate differences in gene expression were observed, primarily in genes related to carbohydrate metabolism and cell wall remodeling. These genes include enzymes of the glyoxylate cycle—malate synthase (ARB_07638) and isocitrate lyase (ARB_07814), and cell wall synthesis associated enzyme 1,3-beta-glucanosyltransferase (ARB_05770) and class III chitinase *chiA2* (ARB_03514). In all cases, *T.benhamiae* var. *benhamiae* formed a distinct cluster from the other taxa. Additionally, *T.japonicum* showed unique expression of polyketide synthase ARB_07994 from cluster A (Fig. 6a).

### Differential expression under *ex vivo* conditions

Under *ex vivo* conditions on MSE, distinct expression profiles were observed among taxa, especially for genes involved in secondary metabolism. Epidemic strains of *T.benhamiae* var. *luteum* showed differential expression of a Fasciclin domain protein (ARB_07991, cluster A) and polyketide synthase ARB_07534 (cluster B). Furthermore, *T.europaeum* displayed significantly lower expression of 1,3-β-glucanosyltransferase (ARB_05770) compared with other taxa (Fig. 6b).

### Evaluation of the *ex vivo* skin model

**Fig 7:**
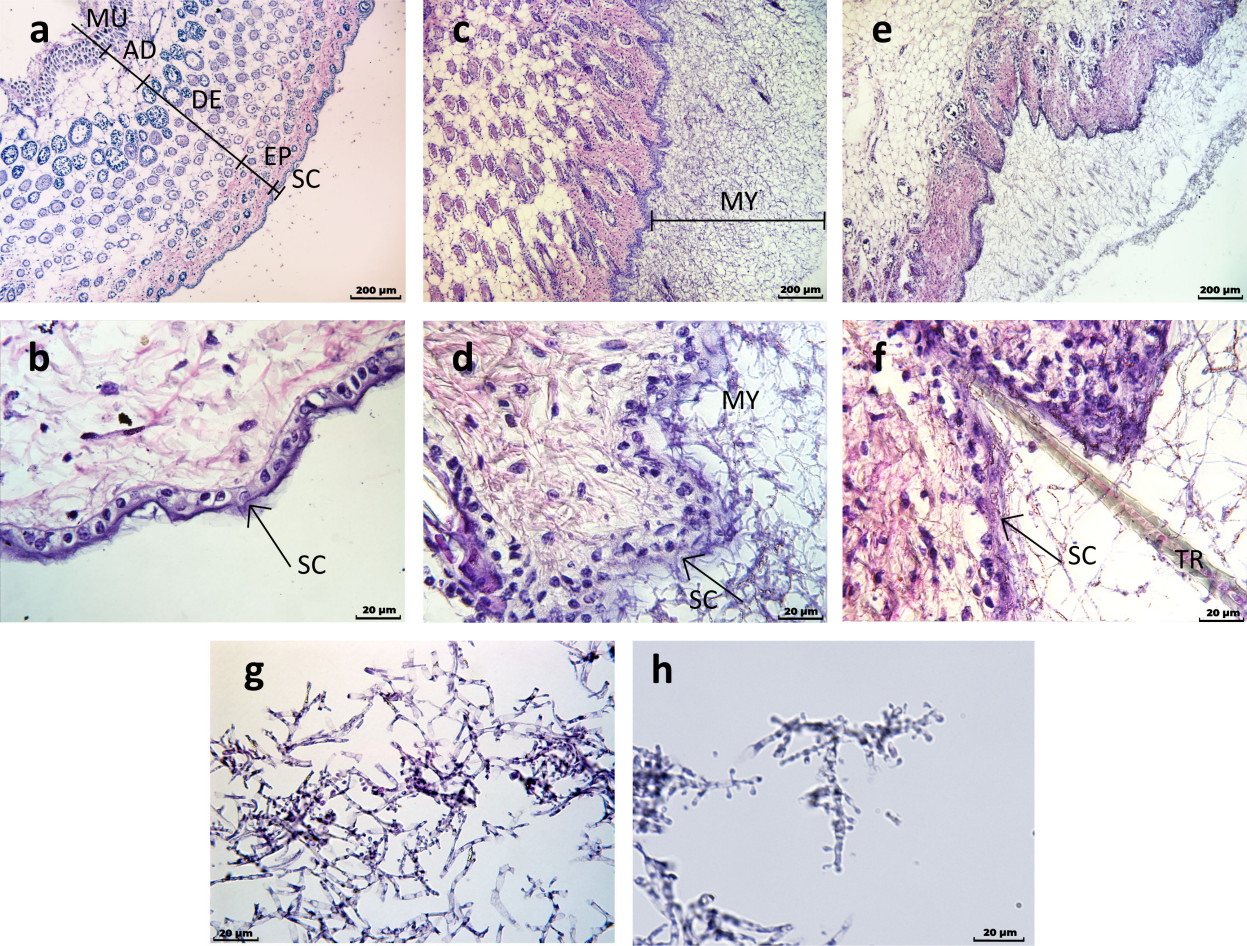
Light microscopy of murine skin explants. (**a and b**) Control (non-inoculated) skin; (**c and d**) skin colonized by *T.benhamiae* var. *luteum* (IHEM 25077); (**e and f**) skin colonized by *T.japonicum* (VUT 97010). (**g and h**) Sporulation observed on the upper layers of mycelium in IHEM 25077 and VUT 97010, likely as a surface-associated response during extended colonization. Skin layers are labeled: SC, stratum corneum; EP, epidermis; DE, dermis; AD, adipose tissue; MU, muscle; TR, trichome. Mycelial layer is marked as MY.

To assess the colonization performance of the strains in the ex vivo model, histological analysis of skin samples was performed. Microscopic examination revealed extensive fungal colonization and disruption of the stratum corneum (Fig. 7c through f). Notably, *T.benhamiae* var. *luteum* produced microconidia in skin tissue, despite showing limited sporulation on agar media (Fig. 7g and h).

## DISCUSSION

In this study, we used a comparative model of four closely related taxa within the *Trichophyton benhamiae* complex (*T.europaeum*, *T.japonicum*, *T.benhamiae* var. *luteum*, and *T.benhamiae* var. *benhamiae*). By examining the differences between these closely related taxa, we aimed to identify the factors influencing their varying abilities to adapt and spread in the host population. Similar comparative transcriptomic approaches have been applied to other fungal pathogens to reveal virulence-associated traits (82, 83). However, studies on dermatophytes have largely focused on comparisons among phylogenetically distant species (84). The use of closely related taxa in our model allowed us to detect subtle but biologically significant differences in gene regulation and adaptation.

A key finding of this study was the upregulation of genes involved in secondary metabolism, particularly in the epidemic taxon *T.benhamiae* var. *luteum*. Among the candidate genes identified, several showed consistent differential expression (Table 2), with most belonging to two biosynthetic gene clusters: cluster A, which includes the vioxanthin, xanthomegnin, and viomellein biosynthetic genes (represented by genes ARB_07994 and ARB_07991), and cluster B, an uncharacterized cluster (represented by gene ARB_07534) with no close homologs. These results, based on RT-qPCR analysis across multiple strains, suggest that secondary metabolite production may contribute to dermatophyte virulence and host adaptation.

The differential expression patterns were not limited to these clusters. The catalase *easC* gene (ARB_04645), which is involved in chanoclavine I biosynthesis, a key precursor in the ergot alkaloid pathway (85, 86), also showed substantial variation in expression across strains, suggesting strain-specific regulation of secondary metabolism. Similarly, the polyketide synthase from cluster A (ARB_07994) was significantly underexpressed in *T.japonicum* under ex vivo conditions (Fig. 4), suggesting environmentally regulated control of polyketide biosynthesis.

Dermatophyte genomes encode more BGCs than other pathogenic fungi (67), yet few have been functionally characterized. Several of their known products—such as vioxanthin, xanthomegnin, and neosartoricin B—are known to have cytotoxic or immunomodulatory effects (18, 86–88). Previous work (89) has shown that secondary metabolite profiles correlate with phylogeny in dermatophytes, indicating that secondary metabolism is under strong evolutionary pressure. The same study identified a distinct group of unique secondary metabolites, particularly sulfur-containing compounds, produced by members of the *T.benhamiae* complex. Our transcriptomic data further support this view, particularly in the case of sulfur-associated metabolism, where multiple genes showed taxon-specific expression (Table 2). These findings suggest that secondary metabolism may shape ecological adaptation and host-pathogen interactions in the *T.benhamiae* complex.

In addition to secondary metabolism, we assessed expression of genes encoding secreted proteases, known virulence factors in dermatophytes. In agreement with earlier findings (23, 24), genes, such as *SUB3* (ARB_00701), *DPPIV* (ARB_06110), *DPPV* (ARB_06651), and *LAP2* (ARB_00494), were upregulated under *ex vivo* conditions. However, their expression was largely uniform across taxa, except for *DPPIV*, which showed some variation. This suggests that secreted proteases may be core virulence determinants, but not discriminatory markers of virulence variation among closely related taxa.

We also observed upregulation of genes involved in carbohydrate and fatty acid metabolism, such as isocitrate lyase (ARB_07814), under *ex vivo* conditions. These genes likely reflect adaptation to host-derived substrates, such as keratin and lipids, which are absent in synthetic media.

Gene expression differences were more pronounced under *ex vivo* conditions than *in vitro*, reinforcing the biological relevance of the model. Strains grown *ex vivo* clustered by taxon (Fig. 3), likely reflecting stronger selective pressures in host-mimicking conditions. This underscores the adaptability of dermatophytes in complex environments, where metabolic flexibility is essential for survival. These transcriptional responses likely reflect underlying host–fungus interactions within the skin tissue, which we were only able to assess at a basic level using histological staining. While H&E staining confirmed fungal invasion into skin tissue, it does not provide detailed resolution of fungal–host interactions at the cellular or ultrastructural level. Future studies could benefit from using complementary imaging approaches, such as fluorescent or electron microscopy, to better characterize the spatial dynamics of colonization. Although the *ex vivo* model is limited by the absence of an active immune response and the use of murine rather than human tissue, it effectively recapitulated differential gene expression and provided a host-mimicking context for assessing fungal adaptation. In contrast, gene expression patterns were highly similar across taxa under *in vitro* conditions, suggesting that species-specific responses are triggered by environmental factors present only *ex vivo*, such as the structural complexity of skin and its derivatives.

An interesting deviation from expected clustering patterns was observed in strain ME 192/12, which grouped with *T.japonicum* despite being genetically assigned to *T.europaeum*. This may reflect taxonomic ambiguity or gene flow between these closely related species (4). Moreover, expression of cell wall remodeling genes, including *chiA2* (ARB_03514) and 1,3-β-glucanosyltransferase (ARB_05770), showed taxon-specific differences. These enzymes play a critical role in fungal cell wall restructuring, potentially contributing to differences in growth morphology and environmental adaptation (90).

Our study provides new insights into the potential virulence factors within the *T.benhamiae* complex. While secreted proteases are traditionally viewed as major contributors to virulence, their expression showed little variation among taxa. In contrast, secondary metabolism genes displayed notable differential expression, particularly under *ex vivo* conditions, underscoring the potential role of secondary metabolites in dermatophyte adaptation and pathogenicity. The ability of these fungi to produce unique compounds, including sulfur-containing metabolites, suggests that secondary metabolic pathways are not only shaped by strong evolutionary pressure but may also be essential for host adaptation and survival.

While our data show consistent differential expression of candidate genes, we acknowledge that variation in gene expression under *ex vivo* conditions may partly reflect differences in fungal biomass among strains. However, the epidemic taxon *T.benhamiae* var. *luteum*, which showed the highest expression of several genes, is known to grow more slowly than the other taxa tested. As a result, it likely produced the lowest fungal biomass in the *ex vivo* model. This supports the interpretation that the observed overexpression reflects true upregulation rather than biomass-driven effects. Furthermore, the use of two validated, stably expressed housekeeping genes (*rpb2* and *adp-rf*) as internal controls for normalization helped reduce bias due to variable fungal loads. Nonetheless, future studies incorporating quantitative fungal burden measurements would help disentangle biomass effects from transcriptional regulation.

Future work should focus on functional validation of genes such as *ARB_07534*, along with broader comparative studies to better characterize the biosynthetic capacity of dermatophytes. This may include LC-MS-based metabolomic profiling of secondary metabolites or targeted gene disruption (e.g., CRISPR-Cas9 knockout of ARB_07534) to confirm their role in virulence and adaptation. Such research will be essential for advancing targeted diagnostics, antifungal strategies, and our understanding of dermatophyte pathogenesis.

## Data Availability

The raw RNA sequencing data generated in this study will be deposited in the NCBI Sequence Read Archive (SRA) under the BioProject accesion number PRJNA1328479. Processed gene expression data and RT-qPCR results (including ΔCt and 2^–ΔΔCt^ values) are available in Supplemental material 3 and 4. Additional data supporting the findings—such as primer sequences, gene annotations, and statistical outputs—are provided in the Supplementary Information or are available from the corresponding author upon reasonable request. All fungal strains used in this study are publicly available through the Culture Collection of Fungi (CCF), Charles University, Prague.

## SUPPLEMENTAL MATERIAL

Supplemental material 1 spectrum.01383-25-s0001.xlsx RNAseq differential expression analysis (DESeq2) comparing *T.benhamiae* var. *luteum* and *T.japonicum*.

Supplemental material 2 spectrum.01383-25-s0002.xlsx Primer sequences and evaluation of reference gene stability.

Supplemental material 3 spectrum.01383-25-s0003.xlsx Results of the RT-qPCR analysis, including relative gene expression (ΔCt) values.

Supplemental material 4 spectrum.01383-25-s0004.xlsx Statistical analysis of RT-qPCR data.

Supplemental material 5 spectrum.01383-25-s0005.xlsx BLAST comparison of secondary metabolite cluster genes between *T. rubrum* and *T. europaeum*.

ASM does not own the copyrights to Supplemental Material that may be linked to, or accessed through, an article. The authors have granted ASM a non-exclusive, world-wide license to publish the Supplemental Material files. Please contact the corresponding author directly for reuse.

## References

[1] Havlickova B, Czaika VA, Friedrich M. 2008. Epidemiological trends in skin mycoses worldwide. Mycoses 51 Suppl 4:2–15. doi:10.1111/j.1439-0507.2008.01606.x 18783559

[2] Shen JJ, Arendrup MC, Verma S, Saunte DML. 2022. The emerging terbinafine-resistant Trichophyton epidemic: what is the role of antifungal susceptibility testing? Dermatology 238:60–79. doi:10.1159/000515290 34058736

[3] Čmoková A, Rezaei-Matehkolaei A, Kuklová I, Kolařík M, Shamsizadeh F, Ansari S, Gharaghani M, Miňovská V, Najafzadeh MJ, Nouripour-Sisakht S, Yaguchi T, Zomorodian K, Zarrinfar H, Hubka V. 2021. Discovery of new Trichophyton members, T. persicum and T. spiraliforme spp. nov., as a cause of highly inflammatory tinea cases in Iran and Czechia. Microbiol Spectr 9:e0028421. doi:10.1128/Spectrum.00284-21 34468188 PMC8557871

[4] Čmoková A, Kolařík M, Dobiáš R, Hoyer LL, Janouškovcová H, Kano R, Kuklová I, Lysková P, Machová L, Maier T, Mallátová N, Man M, Mencl K, Nenoff P, Peano A, Prausová H, Stubbe D, Uhrlaß S, Větrovský T, Wiegand C, Hubka V. 2020. Resolving the taxonomy of emerging zoonotic pathogens in the Trichophyton benhamiae complex. Fungal Divers 104:333–387. doi:10.1007/s13225-020-00465-3

[5] Nenoff P, Uhrlaß S, Krüger C, Erhard M, Hipler UC, Seyfarth F, Herrmann J, Wetzig T, Schroedl W, Gräser Y. 2014. Trichophyton species of Arthroderma benhamiae - a new infectious agent in dermatology. J Dtsch Dermatol Ges 12:571–581. doi:10.1111/ddg.12390 24981469

[6] Bartosch T, Frank A, Günther C, Uhrlaß S, Heydel T, Nenoff P, Baums CG, Schrödl W. 2019. Trichophyton benhamiae and T. mentagrophytes target guinea pigs in a mixed small animal stock. Med Mycol Case Rep 23:37–42. doi:10.1016/j.mmcr.2018.11.005 30560049 PMC6290094

[7] Berlin M, Kupsch C, Ritter L, Stoelcker B, Heusinger A, Gräser Y. 2020. German-wide analysis of the prevalence and the propagation factors of the zoonotic dermatophyte Trichophyton benhamiae. JoF 6:161. doi:10.3390/jof6030161 32899171 PMC7558194

[8] Monod M. 2008. Secreted proteases from dermatophytes. Mycopathologia 166:285–294. doi:10.1007/s11046-008-9105-4 18478360

[9] Vermout S, Baldo A, Tabart J, Losson B, Mignon B. 2008. Secreted dipeptidyl peptidases as potential virulence factors for microsporum canis. FEMS Immunol Med Microbiol 54:299–308. doi:10.1111/j.1574-695X.2008.00479.x 19049642

[10] Burstein VL, Beccacece I, Guasconi L, Mena CJ, Cervi L, Chiapello LS. 2020. Skin immunity to dermatophytes: from experimental infection models to human disease. Front Immunol 11:605644. doi:10.3389/fimmu.2020.605644 33343578 PMC7738607

[11] Matousek JL, Campbell KL. 2002. A comparative review of cutaneous pH. Vet Dermatol 13:293–300. doi:10.1046/j.1365-3164.2002.00312.x 12464061

[12] Dahl MV. 1993. Suppression of immunity and inflammation by products produced by dermatophytes. J Am Acad Dermatol 28:S19–S23. doi:10.1016/S0190-9622(09)80303-4 8496406

[13] Kar B, Patel P, Free SJ. 2019. Trichophyton rubrum LysM proteins bind to fungal cell wall chitin and to the N-linked oligosaccharides present on human skin glycoproteins. PLoS One 14:e0215034. doi:10.1371/journal.pone.0215034 30947244 PMC6449025

[14] Martinez-Rossi NM, Persinoti GF, Peres NTA, Rossi A. 2012. Role of pH in the pathogenesis of dermatophytoses. Mycoses 55:381–387. doi:10.1111/j.1439-0507.2011.02162.x 22211778

[15] Bitencourt TA, Neves-da-Rocha J, Martins MP, Sanches PR, Lang EAS, Bortolossi JC, Rossi A, Martinez-Rossi NM. 2021. StuA-Regulated processes in the dermatophyte Trichophyton rubrum: transcription profile, cell-cell adhesion, and immunomodulation. Front Cell Infect Microbiol 11:643659. doi:10.3389/fcimb.2021.643659 34169004 PMC8218993

[16] Maranhão FCA, Paião FG, Martinez-Rossi NM. 2007. Isolation of transcripts over-expressed in human pathogen Trichophyton rubrum during growth in keratin. Microb Pathog 43:166–172. doi:10.1016/j.micpath.2007.05.006 17590307

[17] Wirth JC, Beesley TE, Anand SR. 1965. The isolation of xanthomegnin from several strains of the dermatophyte, Trichophyton rubrum. Phytochemistry 4:505–509. doi:10.1016/S0031-9422(00)86204-4

[18] Ng AS, Just G, Blank F. 1969. Metabolites of pathogenic fungi. VII. On the structure and stereochemistry of xanthomegnin, vioxanthin, and viopurpurin, pigments from Trichophyton violaceum. Can J Chem 47:1223–1227. doi:10.1139/v69-197

[19] Youngchim S, Pornsuwan S, Nosanchuk JD, Dankai W, Vanittanakom N. 2011. Melanogenesis in dermatophyte species in vitro and during infection. Microbiology (Reading) 157:2348–2356. doi:10.1099/mic.0.047928-0 21565930 PMC3167886

[20] Lappin-Scott HM, Rogers ME, Adlard MW, Holt G, Noble WC. 1985. High-performance liquid chromatographic identification of beta-lactam antibiotics produced by dermatophytes. J Appl Bacteriol 59:437–441. doi:10.1111/j.1365-2672.1985.tb03343.x 3878840

[21] Kandemir H, Dukik K, Hagen F, Ilkit M, Gräser Y, de Hoog GS. 2020. Polyphasic discrimination of Trichophyton tonsurans and T. equinum from humans and horses. Mycopathologia 185:113–122. doi:10.1007/s11046-019-00344-9 31278475

[22] Martinez DA, Oliver BG, Gräser Y, Goldberg JM, Li W, Martinez-Rossi NM, Monod M, Shelest E, Barton RC, Birch E, et al.. 2012. Comparative genome analysis of Trichophyton rubrum and related dermatophytes reveals candidate genes involved in infection. mBio 3:e00259-12. doi:10.1128/mBio.00259-12 22951933 PMC3445971

[23] Staib P, Zaugg C, Mignon B, Weber J, Grumbt M, Pradervand S, Harshman K, Monod M. 2010. Differential gene expression in the pathogenic dermatophyte Arthroderma benhamiae in vitro versus during infection. Microbiology (Reading) 156:884–895. doi:10.1099/mic.0.033464-0 19942661

[24] Tran VDT, De Coi N, Feuermann M, Schmid-Siegert E, Băguţ E-T, Mignon B, Waridel P, Peter C, Pradervand S, Pagni M, Monod M. 2016. RNA sequencing-based genome reannotation of the dermatophyte Arthroderma benhamiae and characterization of its secretome and whole gene expression profile during Infection. mSystems 1:00036–16. doi:10.1128/mSystems.00036-16 PMC5069957 27822542

[25] Maciel Quatrin P, Flores Dalla Lana D, Andrzejewski Kaminski TF, Meneghello Fuentefria A. 2019. Fungal infection models: current progress of ex vivo methods. Mycoses 62:860–873. doi:10.1111/myc.12961 31271676

[26] Tabart J, Baldo A, Vermout S, Nusgens B, Lapiere C, Losson B, Mignon B. 2007. Reconstructed interfollicular feline epidermis as a model for Microsporum canis dermatophytosis. J Med Microbiol 56:971–975. doi:10.1099/jmm.0.47115-0 17577064

[27] Baumbach C-M, Schrödl W, Nenoff P, Uhrlaß S, Mülling CKW, Michler JK. 2020. Modeling dermatophytosis: Guinea pig skin explants represent a highly suitable model to study Trichophyton benhamiae infections. J Dermatol 47:8–16. doi:10.1111/1346-8138.15150 31782188

[28] Baumbach C-M, Michler JK, Nenoff P, Uhrlaß S, Schrödl W. 2020. Visualising virulence factors: Trichophyton benhamiaes subtilisins demonstrated in a guinea pig skin ex vivo model. Mycoses 63:970–978. doi:10.1111/myc.13136 32620041

[29] Ho F-H, Delgado-Charro MB, Bolhuis A. 2020. Evaluation of an explanted porcine skin model to investigate infection with the dermatophyte Trichophyton rubrum. Mycopathologia 185:233–243. doi:10.1007/s11046-020-00438-9 32108288

[30] Corzo-León DE, Munro CA, MacCallum DM. 2019. An ex vivo human skin model to study superficial fungal infections. Front Microbiol 10:1172. doi:10.3389/fmicb.2019.01172 31231322 PMC6560176

[31] Duek L, Kaufman G, Ulman Y, Berdicevsky I. 2004. The pathogenesis of dermatophyte infections in human skin sections. J Infect 48:175–180. doi:10.1016/j.jinf.2003.09.008 14720494

[32] Poumay Y, Dupont F, Marcoux S, Leclercq-Smekens M, Hérin M, Coquette A. 2004. A simple reconstructed human epidermis: preparation of the culture model and utilization in in vitro studies. Arch Dermatol Res 296:203–211. doi:10.1007/s00403-004-0507-y 15349789

[33] Peled IJ, Notea E, Lindenbaum E. 1991. Prolonged skin graft preservation with keratinocyte culture medium. Eur J Plast Surg 14:232–234. doi:10.1007/BF00176637

[34] Byun T, Kofod L, Blinkovsky A. 2001. Synergistic action of an X-prolyl dipeptidyl aminopeptidase and a non-specific aminopeptidase in protein hydrolysis. J Agric Food Chem 49:2061–2063. doi:10.1021/jf001091m 11308367

[35] Dobin A, Davis CA, Schlesinger F, Drenkow J, Zaleski C, Jha S, Batut P, Chaisson M, Gingeras TR. 2013. STAR: ultrafast universal RNA-seq aligner. Bioinformatics 29:15–21. doi:10.1093/bioinformatics/bts635 23104886 PMC3530905

[36] Langmead B, Salzberg SL. 2012. Fast gapped-read alignment with Bowtie 2. Nat Methods 9:357–359. doi:10.1038/nmeth.1923 22388286 PMC3322381

[37] Love MI, Huber W, Anders S. 2014. Moderated estimation of fold change and dispersion for RNA-seq data with DESeq2. Genome Biol 15:550. doi:10.1186/s13059-014-0550-8 25516281 PMC4302049

[38] Zaugg C, Jousson O, Léchenne B, Staib P, Monod M. 2008. Trichophyton rubrum secreted and membrane-associated carboxypeptidases. Int J Med Microbiol 298:669–682. doi:10.1016/j.ijmm.2007.11.005 18222721

[39] Xie F, Xiao P, Chen D, Xu L, Zhang B. 2012. miRDeepFinder: a miRNA analysis tool for deep sequencing of plant small RNAs. Plant Mol Biol 80. doi:10.1007/s11103-012-9885-2 22290409

[40] Pfaffl MW, Tichopad A, Prgomet C, Neuvians TP. 2004. Determination of stable housekeeping genes, differentially regulated target genes and sample integrity: BestKeeper--Excel-based tool using pair-wise correlations. Biotechnol Lett 26:509–515. doi:10.1023/b:bile.0000019559.84305.47 15127793

[41] Andersen CL, Jensen JL, Ørntoft TF. 2004. Normalization of real-time quantitative reverse transcription-PCR data: a model-based variance estimation approach to identify genes suited for normalization, applied to bladder and colon cancer data sets. Cancer Res 64:5245–5250. doi:10.1158/0008-5472.CAN-04-0496 15289330

[42] Vandesompele J, De Preter K, Pattyn F, Poppe B, Van Roy N, De Paepe A, Speleman F. 2002. Accurate normalization of real-time quantitative RT-PCR data by geometric averaging of multiple internal control genes. Genome Biol 3:1–12. doi:10.1186/gb-2002-3-7-research0034 PMC126239 12184808

[43] Jacob TR, Peres NTA, Persinoti GF, Silva LG, Mazucato M, Rossi A, Martinez-Rossi NM. 2012. Rpb2 is a reliable reference gene for quantitative gene expression analysis in the dermatophyte Trichophyton rubrum. Med Mycol 50:368–377. doi:10.3109/13693786.2011.616230 21958376

[44] Ciesielska Anita, Stączek P. 2018. Selection and validation of reference genes for qRT-PCR analysis of gene expression in Microsporum canis growing under different adhesion-inducing conditions. Sci Rep 8:1197. doi:10.1038/s41598-018-19680-9 29352152 PMC5775245

[45] Ciesielska A, Oleksak B, Stączek P. 2019. Reference genes for accurate evaluation of expression levels in Trichophyton interdigitale grown under different carbon sources, pH levels and phosphate levels. Sci Rep 9:5566. doi:10.1038/s41598-019-42065-5 30944363 PMC6447595

[46] Zaugg C, Monod M, Weber J, Harshman K, Pradervand S, Thomas J, Bueno M, Giddey K, Staib P. 2009. Gene expression profiling in the human pathogenic dermatophyte Trichophyton rubrum during growth on proteins. Eukaryot Cell 8:241–250. doi:10.1128/EC.00208-08 19098130 PMC2643602

[47] Ganger MT, Dietz GD, Ewing SJ. 2017. A common base method for analysis of qPCR data and the application of simple blocking in qPCR experiments. BMC Bioinformatics 18:534. doi:10.1186/s12859-017-1949-5 29191175 PMC5709943

[48] R Core Team. 2022 R: a language and environment for statistical computing. Available from: https://www.R-project.org

[49] Wickham H. 2007. Reshaping data with the reshape package. J Stat Softw 21:1–20. doi:10.18637/jss.v021.i12

[50] Lê S, Josse J, Husson F. 2008. FactoMineR: an R package for multivariate analysis. J Stat Softw 25:1–18. doi:10.18637/jss.v025.i01

[51] Kassambara A, factoextra MF. 2020 R package version 1.0.7. Extract and Visualize the Results of Multivariate Data Analyses. Available from: https://CRAN.R-project.org/package=factoextra

[52] Wickham H, Averick M, Bryan J, Chang W, McGowan L, François R, Grolemund G, Hayes A, Henry L, Hester J, Kuhn M, Pedersen T, Miller E, Bache S, Müller K, Ooms J, Robinson D, Seidel D, Spinu V, Takahashi K, Vaughan D, Wilke C, Woo K, Yutani H. 2019. Welcome to the Tidyverse. JOSS 4:1686. doi:10.21105/joss.01686

[53] Valero-Mora PM. 2010. Ggplot2: elegant graphics for data analysis. J Stat Softw 35:212. doi:10.18637/jss.v035.b01

[54] Garnier S, Ross N, Rudis R, Camargo AP, Sciaini M, Scherer C. 2021 viridis: Colorblind friendly color maps for R. R package version 0.6.2. https://CRAN.R-project.org/package=viridis.

[55] Auguie B. 2017 R package version 2.3. gridExtra: Miscellaneous Functions for “Grid” Graphics. Available from: https://CRAN.R-project.org/package=gridExtra

[56] Wilke C. 2020 R Package Version 1.1.1. cowplot:Streamlined Plot Theme and Plot Annotations for “Ggplot2.” Available from: https://CRAN.R-project.org/package=cowplot

[57] Blin K, Shaw S, Augustijn HE, Reitz ZL, Biermann F, Alanjary M, Fetter A, Terlouw BR, Metcalf WW, Helfrich EJN, van Wezel GP, Medema MH, Weber T. 2023. antiSMASH 7.0: new and improved predictions for detection, regulation, chemical structures and visualisation. Nucleic Acids Res 51:W46–W50. doi:10.1093/nar/gkad344 37140036 PMC10320115

[58] Altschul SF, Gish W, Miller W, Myers EW, Lipman DJ. 1990. Basic local alignment search tool. J Mol Biol 215:403–410. doi:10.1016/S0022-2836(05)80360-2 2231712

[59] Katoh K, Rozewicki J, Yamada KD. 2019. MAFFT online service: multiple sequence alignment, interactive sequence choice and visualization. Brief Bioinform 20:1160–1166. doi:10.1093/bib/bbx108 28968734 PMC6781576

[60] Wilkins D. 2023 R package version 0.5.1. gggenes: draw gene arrow maps in “ggplot2.” Available from: https://CRAN.R-project.org/package=gggenes

[61] Blum F. 1893. Der formaldehyde als hartungsmittle. Microsc Acta 10:314–315.

[62] Mayer P. 1896. No Title, p 303. In Mitt. zool. Stn. Neapel

[63] Lillie RD. 1965. Histopathologic technic and practical histochemistry. 3rd ed. McGraw-Hill Book Co, New York.

[64] Avwioro G. 2011. Histochemical uses of haematoxylin - a review. Jpcs 1:24–34.

[65] Wallwey C, Heddergott C, Xie X, Brakhage AA, Li SM. 2012. Genome mining reveals the presence of a conserved gene cluster for the biosynthesis of ergot alkaloid precursors in the fungal family Arthrodermataceae. Microbiology (Reading, Engl) 158:1634–1644. doi:10.1099/mic.0.056796-0 22403186

[66] Descamps F, Brouta F, Monod M, Zaugg C, Baar D, Losson B, Mignon B. 2002. Isolation of a Microsporum canis gene family encoding three subtilisin-like proteases expressed in vivo*.* J Invest Dermatol 119:830–835. doi:10.1046/j.1523-1747.2002.01784.x 12406327

[67] Baldo A, Mathy A, Tabart J, Camponova P, Vermout S, Massart L, Maréchal F, Galleni M, Mignon B. 2010. Secreted subtilisin Sub3 from Microsporum canis is required for adherence to but not for invasion of the epidermis. Br J Dermatol 162:990–997. doi:10.1111/j.1365-2133.2009.09608.x 19995373

[68] Burmester A, Shelest E, Glöckner G, Heddergott C, Schindler S, Staib P, Heidel A, Felder M, Petzold A, Szafranski K, et al.. 2011. Comparative and functional genomics provide insights into the pathogenicity of dermatophytic fungi. Genome Biol 12:R7. doi:10.1186/gb-2011-12-1-r7 21247460 PMC3091305

[69] Monod M, Léchenne B, Jousson O, Grand D, Zaugg C, Stöcklin R, Grouzmann E. 2005. Aminopeptidases and dipeptidyl-peptidases secreted by the dermatophyte Trichophyton rubrum. Microbiology (Reading) 151:145–155. doi:10.1099/mic.0.27484-0 15632434

[70] Kaufman G, Berdicevsky I, Woodfolk JA, Horwitz BA. 2005. Markers for host-induced gene expression in Trichophyton dermatophytosis. Infect Immun 73:6584–6590. doi:10.1128/IAI.73.10.6584-6590.2005 16177334 PMC1230929

[71] Bateman A. 2019. UniProt: a worldwide hub of protein knowledge. Nucleic Acids Res 47:D506–D515. doi:10.1093/nar/gky1049 30395287 PMC6323992

[72] Kornberg HL, Krebs HA. 1957. Synthesis of cell constituents from C2-units by a modified tricarboxylic acid cycle. Nature 179:988–991. doi:10.1038/179988a0 13430766

[73] Grumbt M, Defaweux V, Mignon B, Monod M, Burmester A, Wöstemeyer J, Staib P. 2011. Targeted gene deletion and in vivo analysis of putative virulence gene function in the pathogenic dermatophyte Arthroderma benhamiae. Eukaryot Cell 10:842–853. doi:10.1128/EC.00273-10 21478433 PMC3127675

[74] Mouyna I, Fontaine T, Vai M, Monod M, Fonzi WA, Diaquin M, Popolo L, Hartland RP, Latgé JP. 2000. Glycosylphosphatidylinositol-anchored glucanosyltransferases play an active role in the biosynthesis of the fungal cell wall. J Biol Chem 275:14882–14889. doi:10.1074/jbc.275.20.14882 10809732

[75] Chen W, Lee MK, Jefcoate C, Kim SC, Chen F, Yu JH. 2014. Fungal cytochrome P450 monooxygenases: their distribution, structure, functions, family expansion, and evolutionary origin. Genome Biol Evol 6:1620–1634. doi:10.1093/gbe/evu132 24966179 PMC4122930

[76] Nielsen CAF, Folly C, Hatsch A, Molt A, Schröder H, O’Connor SE, Naesby M. 2014. The important ergot alkaloid intermediate chanoclavine-I produced in the yeast Saccharomyces cerevisiae by the combined action of EasC and EasE from Aspergillus japonicus*.* Microb Cell Fact 13:95. doi:10.1186/s12934-014-0095-2 25112180 PMC4249865

[77] Fürtges L, Obermaier S, Thiele W, Foegen S, Müller M. 2019. Diversity in fungal intermolecular phenol coupling of polyketides: regioselective laccase-based systems. Chembiochem 20:1928–1932. doi:10.1002/cbic.201900041 30868712

[78] Ninomiya A, Masuda K, Yamada T, Kuroki M, Ban S, Yaguchi T, Urayama SI, Hagiwara D. 2025. Rediscovery of viomellein as an antibacterial compound and identification of its biosynthetic gene cluster in dermatophytes. Appl Environ Microbiol 91:e0243124. doi:10.1128/aem.02431-24 40197033 PMC12093964

[79] Fraser JA, Davis MA, Hynes MJ. 2002. A gene from Aspergillus nidulans with similarity to URE2 of Saccharomyces cerevisiae encodes a glutathione S-transferase which contributes to heavy metal and xenobiotic resistance. Appl Environ Microbiol 68:2802–2808. doi:10.1128/AEM.68.6.2802-2808.2002 12039735 PMC123945

[80] Hendrickson L, Ray Davis C, Roach C, Kim Nguyen D, Aldrich T, McAda PC, Reeves CD. 1999. Lovastatin biosynthesis in Aspergillus terreus: characterization of blocked mutants, enzyme activities and a multifunctional polyketide synthase gene. Chemistry & Biology 6:429–439. doi:10.1016/S1074-5521(99)80061-1 10381407

[81] Yin WB, Chooi YH, Smith AR, Cacho RA, Hu Y, White TC, Tang Y. 2013. Discovery of cryptic polyketide metabolites from dermatophytes using heterologous expression in Aspergillus nidulans. ACS Synth Biol 2:629–634. doi:10.1021/sb400048b 23758576 PMC3795930

[82] Gu X, Yang S, Yang X, Yao L, Gao X, Zhang M, Liu W, Zhao H, Wang Q, Li Z, Li Z, Ding J. 2020. Comparative transcriptome analysis of two Cercospora sojina strains reveals differences in virulence under nitrogen starvation stress. BMC Microbiol 20:166. doi:10.1186/s12866-020-01853-0 32546122 PMC7298872

[83] Gai X, Li S, Jiang N, Sun Q, Xuan YH, Xia Z. 2022. Comparative transcriptome analysis reveals that ATP synthases regulate Fusarium oxysporum virulence by modulating sugar transporter gene expressions in tobacco. Front Plant Sci 13:978951. doi:10.3389/fpls.2022.978951 36061782 PMC9433920

[84] Chen B, Zhang J, Li J, Qian Y, Huang B, Wu X. 2024. Comparative transcriptome analysis of T. rubrum, T. mentagrophytes, and M. gypseum dermatophyte biofilms in response to photodynamic therapy. Mycopathologia 189:59. doi:10.1007/s11046-024-00865-y 38890181

[85] Goetz KE, Coyle CM, Cheng JZ, O’Connor SE, Panaccione DG. 2011. Ergot cluster-encoded catalase is required for synthesis of chanoclavine-I in Aspergillus fumigatus. Curr Genet 57:201–211. doi:10.1007/s00294-011-0336-4 21409592

[86] Delucca AJ, Dunn JJ, Lee LS, Ciegler A. 1982. Toxicity, mutagenicity and teratogenicity of brevianamide, viomellein and xanthomegnin; secondary metabolites of Penicillium viridicatum . J Food Saf 4:165–168. doi:10.1111/j.1745-4565.1982.tb00440.x

[87] Yang X-Y, Cai S-X, Zhang W-J, Tang X-L, Shin H-Y, Lee J-Y, Gu Q-Q, Park H. 2008. Semi-vioxanthin isolated from marine-derived fungus regulates tumor necrosis factor-alpha, cluster of differentiation (CD) 80, CD86, and major histocompatibility complex class II expression in RAW264.7 cells via nuclear factor-kappaB and mitogen-activated protein kinase signaling pathways. Biol Pharm Bull 31:2228–2233. doi:10.1248/bpb.31.2228 19043204

[88] Chooi YH, Fang J, Liu H, Filler SG, Wang P, Tang Y. 2013. Genome mining of a prenylated and immunosuppressive polyketide from pathogenic fungi. Org Lett 15:780–783. doi:10.1021/ol303435y 23368997 PMC3576815

[89] Machová L, Gaida M, Semerád J, Kolařík M, Švarcová M, Jašica A, Grasserová A, Awokunle Hollá S, Hubka V, Stefanuto PH, Cajthaml T, Focant JF, Wennrich A. 2025. First step on the way to identify dermatophytes using odour fingerprints. Mycopathologia 190:10. doi:10.1007/s11046-024-00905-7 39775995 PMC11706917

[90] Adams DJ. 2004. Fungal cell wall chitinases and glucanases. Microbiology (Reading) 150:2029–2035. doi:10.1099/mic.0.26980-0 15256547

